# Some bounds on the distance-sum-connectivity matrix

**DOI:** 10.1186/s13660-018-1766-z

**Published:** 2018-07-13

**Authors:** Gülistan Kaya Gök

**Affiliations:** Department of Mathematics Education, Hakkari University, Hakkari, Turkey

**Keywords:** 05C22, 05C50, Distance-sum-connectivity matrix, Bounds

## Abstract

The distance-sum-connectivity matrix of a graph *G* is expressed by $\delta(i)$ and $\delta(j)$ such that $i,j\in V$. $\delta(i)$ and $\delta(j)$ are represented by a sum of the distance matrices for $i< v$ and $j< v$, respectively.

The purpose of this paper is to give new inequalities involving the eigenvalues, the graph energy, the graph incidence energy, and the matching energy. So, we have some results in terms of the edges, the vertices, and the degrees.

## Introduction

Let *G* be a simple, finite, connected graphs with the vertex set $V(G)$ and the edge set $E(G)$. By $d_{i}$ we denote the degree of a vertex. Throughout this paper, the vertex degrees are assumed to be ordered non-increasingly. The maximum and the minimum vertex degrees in a graph are denoted by Δ and *δ*, respectively. If any vertices *i* and *j* are adjacent, then we use the notation $i\sim j$.

The distance-sum-connectivity matrix is defined by the displacement of the vertex degrees and the distance sum. This matrix is denoted by ^*δ*^*X* and represented in [1–18] by

$$ ^{\delta}\mathit{X}= \textstyle\begin{cases} (\delta(i)\delta(j))^{\frac{-1}{2}} & \text{if }i\sim j, \\ 0& \text{otherwise.}\end{cases} $$

The distance sum is $\delta(i)=\sum_{j=1}^{v}(^{v}D_{ij})$ such that D is the distance matrix. The distance-sum-connectivity matrix is an interesting matrix, and this paper deals with bounds of this matrix. We find some upper bounds and these bounds contain the edge numbers, the vertex numbers, and the eigenvalues. The eigenvalues of this matrix are $\lambda_{1}(^{\delta}\mathit{X}), \lambda_{2}(^{\delta}\mathit {X}),\ldots,\lambda_{n}(^{\delta}\mathit{X})$ such that $\lambda _{1}(^{\delta}\mathit{X})\geq\lambda_{2}(^{\delta}\mathit{X})\geq \cdots\geq\lambda_{n}(^{\delta}\mathit{X})$. We will accept $\lambda _{1}(^{\delta}\mathit{X})$ as the spectral radius of the graph $^{\delta }\mathit{X}(G)$, and we will take $\lambda_{1}(^{\delta}\mathit{X})$ as $\lambda_{1}$ for convenience. Basic properties of $\lambda_{i}$ are $\sum_{i=1}^{n}\lambda_{i}=0$, $\sum_{i=1}^{n}\lambda_{i}^{2}=2m$, and $\operatorname{det}(^{\delta}\mathit{X})=\prod_{i=1}^{n}\lambda_{i}$. *G* is a regular graph with order *n* if and only if $\lambda_{1}\geq{\frac{2m}{n}}$ [3]. The energy of $(^{\delta}\mathit{X})$ is described as $E(^{\delta }\mathit{X})=\sum_{i=1}^{n}|\lambda_{i}(G)|$. Some properties about the graph energy may be found [7, 10]. The incidence energy *IE* of *G* is introduced by Joojondeh et al. [13] as the sum of singular values of the incidence matrix of *G*. The incidence matrix of a graph *G* is defined as

$$ I(G)=\textstyle\begin{cases} 1& \text{if } v_{i}\text{ is incident with }v_{j}, \\ 0 & \text{otherwise.}\end{cases} $$

The singular values are $q_{1}(^{\delta}\mathit{X})$, $q_{2}(^{\delta }\mathit{X}),\ldots,q_{n}(^{\delta}\mathit{X})$ such that $q_{1}(^{\delta }\mathit{X})\geq q_{2}(^{\delta}\mathit{X})\geq\cdots\geq q_{n}(^{\delta }\mathit{X})$. We use $q_{i}(^{\delta}\mathit{X})$ as $q_{i}$ for brevity. The incidence energy of a graph is represented by $\mathit{IE}=\mathit{IE}(G)=\sum_{i=1}^{n}\sqrt{q_{i}(G)}$. See [8] and [9].

The number of *k*-matchings of a graph *G* is denoted by $m(G,k)$. The matching polynomial of a graph is described by $\alpha(G)=\alpha (G,\lambda)=\sum_{k\geq0}(-1)^{k}m(G,k)\lambda^{n-2k}$ (see [6]).

The matching energy of a graph *G* is defined as

$$ \mathit{ME}=\mathit{ME}(G)=\frac{2}{pi} \int_{o}^{\infty}\frac{1}{x^{2}}\operatorname{ln} \biggl[\sum _{k\geq 0}m(G,k)x^{2k} \biggr]\,dx \quad (\text{see [11]}). $$

The paper is planned as follows. In Sect. 2, we explain previous works. In the next section, we give a survey on upper bound for the first greatest eigenvalue $\lambda_{1}$ and the second greatest eigenvalue $\lambda_{2}$ using the edge number, the vertex number, and the degree. In Sect. 3.2, we focus on the upper bound for energy of $^{\delta}\mathit{X}(G)$ and are concerned with the vertex number, the distance matrix, and the determinant of ^*δ*^*X*. In addition, we deal with some results for the incidence energy of $^{\delta}\mathit{X}(G)$, and we find sharp inequalities of $\mathit{IE}(^{\delta }\mathit{X}(G))$. In Sect. 3.3, we determine different results for the matching energy of a graph with some fixed parameters.

## Preliminaries

In order to achieve our plan, we need the following lemmas and theorems.

### Lemma 2.1

([12])

*Let* $\lambda_{1}(A)$ *be a spectral radius and* $A=(a_{ij})$ *be an irreducible nonnegative matrix with* $R_{i}(A)=\sum_{j=1}^{m}a_{ij}$. *Then*

2.1 $$\begin{aligned} \bigl(\min{R_{i}(A):1\le i \le n} \bigr)\le\lambda_{1}(A) \le \bigl(\max{R_{i}(A):1\le i \le n} \bigr). \end{aligned}$$

### Lemma 2.2

([4])

*If* *G* *is a simple*, *connected graph and* $m_{i}$ *is the average degree of the vertices adjacent to* $v_{i} \in V$, *then*

2.2 $$\begin{aligned} \lambda_{1}(G) \le \max( {\sqrt{m_{i}m_{j}}:1 \le i,j \le n, v_{i},v_{j} \in E}). \end{aligned}$$

Ozeki established Ozeki’s inequality in [16]. This inequality holds some bounds for our graph energy. This inequality is as follows.

### Theorem 2.3

(Ozeki’s inequality)

*If* $a_{i},b_{i}\in R^{+}$, $1\leq i\leq n$, *then*

$$\begin{aligned} \sum_{i=1}^{n}a_{i}^{2} \sum_{i=1}^{n}b_{i}^{2}- \Biggl(\sum_{i=1}^{n}a_{i}b_{i} \Biggr)^{2}\leq\frac{n^{2}}{4}(M_{1}M_{2}-m_{1}m_{2})^{2}, \end{aligned}$$

*where* $M_{1}=\max_{1\leq i\leq n}a_{i}$, $M_{2}=\max_{1\leq i\leq n}b_{i}$, $m_{1}=\min_{1\leq i\leq n}a_{i}$, *and* $m_{2}=\max_{1\leq i\leq n}b_{i}$.

Polya–Szego found an interesting inequality in [17]. This inequality is set as follows.

### Theorem 2.4

(Polya–Szego inequality)

*If* $s_{i},t_{i}\in R^{+}$ *for* $1\leq i\leq n$, *then*

$$\begin{aligned} \sum_{i=1}^{n}s_{i}^{2} \sum_{i=1}^{n}t_{i}^{2} \leq\frac{1}{4} \biggl(\sqrt {\frac{K_{1}K_{2}}{k_{1}k_{2}}}+\sqrt{\frac {k_{1}k_{2}}{K_{1}K_{2}}} \biggr)^{2} \Biggl(\sum_{i=1}^{n}s_{i}t_{i} \Biggr)^{2}, \end{aligned}$$

*where* $K_{1}=\max_{1\leq i\leq n}s_{i}$, $K_{2}=\max_{1\leq i\leq n}t_{i}$, $k_{1}=\min_{1\leq i\leq n}s_{i}$, *and* $k_{2}=\max_{1\leq i\leq n}t_{i}$.

Let *G* be a simple graph and *X* and *Y* be any real symmetric matrices of *G*. Let us consider eigenvalues of these matrices. These eigenvalues hold in the following lemma.

### Lemma 2.5

([5])

*Let* *M* *and* *N* *be two real symmetric matrices and* $1\leq\ell\leq n$, *then*

$$\begin{aligned} \sum_{i=1}^{\ell}\lambda_{i}(M+N) \le\sum_{i=1}^{\ell}\lambda _{i}(M)+ \sum_{i=1}^{\ell}\lambda_{i}(N). \end{aligned}$$

Let $x_{1}, x_{2},\ldots,x_{s}$ be positive real numbers for $1\leq t\leq s$. $M_{t}$ is defined as follows:

$$\begin{aligned} &M_{1}=\frac{x_{1}+x_{2}+\cdots+x_{s}}{s}, \\ &M_{2}=\frac {x_{1}x_{2}+x_{1}x_{3}+\cdots+x_{1}x_{s}+x_{2}x_{3}+\cdots+x_{s-1}x_{s}}{\frac {1}{2}s(s-1)}, \\ &\cdots \\ &M_{s-1}=\frac {x_{1}x_{2}+\cdots+x_{s-1}+x_{1}x_{2}+\cdots+x_{s-2}x_{s}+\cdots+x_{2}x_{3}+\cdots+x_{s-1}x_{s}}{s}, \\ &M_{s}=x_{1}x_{2}\cdots x_{s}. \end{aligned}$$

### Lemma 2.6

(Maclaurin’s symmetric mean inequality [2])

*Let* $x_{1},x_{2},\ldots,x_{s}$ *be real nonnegative numbers*, *then*

$$ M_{1}\geq M_{2}^{\frac{1}{2}}\geq M_{3}^{\frac{1}{3}} \geq\cdots\geq M_{s}^{\frac{1}{s}}. $$

*This equality holds if and only if* $x_{1}=x_{2}=\cdots=x_{s}$.

### Theorem 2.7

*Let* *G* *be a simple graph*. *Let zeros of the matching polynomial of this graph be* $\mu_{1},\mu_{2},\ldots,\mu_{n}$. *Then*

$$\begin{aligned} \mathit{ME}(G)=\sum_{i=1}^{n} \vert \mu_{i} \vert . \end{aligned}$$

*The zeros of the matching polynomial provide the equations* $\sum_{i=1}^{n}\mu_{i}^{2}=2m$ *and* $\sum_{i< j}\mu_{i}\mu_{j}=-m$.

## Main results

### Upper bounds on eigenvalues

A lot of bounds for the eigenvalues have been found. We now establish further bounds for $\lambda_{1}$ and $\lambda_{2}$ involving the *n*, *m* and *d*. Firstly we give some known bounds about graph theory.

In the reference [14] a lower bound is given:

$$\begin{aligned} E(G)\geq2\sqrt{m} \end{aligned}$$

if and only if *G* consists of a complete bipartite graph $K_{x,y}$. In this note, $xy=m$.

Indeed, McClelland’s famous bound is [15] $E(G)\leq\sqrt{2mn}$.

We now will give an upper bound for the eigenvalues of $^{\delta}\mathit{X}(G)$.

#### Theorem 3.1

*If* *G* *is a simple*, *connected graph and* *D* *is the distance matrix of* *G*, *then*

$$\begin{aligned} \lambda_{1}(G) \le \frac{1}{^{4}\sqrt{D_{in}D_{jn}}}. \end{aligned}$$

#### Proof

Let $X=(x_{1},x_{2},\ldots,x_{n})^{T}$ be an eigenvector of $D(G)^{-1}$($^{\delta}\mathit{X}(G)$)$D(G)$. Let one eigencomponent $x_{i}=1$ and the other eigencomponent $0< x_{k}\le1$ for every *k*. Let $x_{j}=\max_{k}({x_{k}: v_{i}v_{k} \in E, i \sim k})$. We know $(D(G)^{-1}$($^{\delta}\mathit{X}(G)$)$D(G))X=\lambda_{1}(G)X$. If we take the *i*th equation of this equation, we obtain

3.1 $$\begin{aligned} \lambda_{1}(G)x_{i}&=\sum_{k} \bigl(\delta(i)\delta(k) \bigr)^{\frac {-1}{2}}x_{k} \end{aligned}$$

3.2 $$\begin{aligned} &=\sum_{k} \Biggl(\sum _{j=1}^{v} \bigl(^{v}D_{ij} \bigr) \sum_{t=1}^{v} \bigl(^{v}D_{kt} \bigr) \Biggr)^{\frac{-1}{2}}x_{k} \end{aligned}$$

3.3 $$\begin{aligned} &=\sum_{k} \biggl(\frac{1}{\sqrt{\sum_{j=1}^{v}(^{v}D_{ij})}} \frac{1}{\sqrt {\sum_{t=1}^{v}(^{v}D_{kt})}} \biggr)x_{k}. \end{aligned}$$

We can take each $D_{ij}$’s as $D_{in}$. So,

3.4 $$\begin{aligned} \lambda_{1}(G)x_{i}\le \biggl(\frac{1}{\sqrt{nD_{in}}}\sum _{k} \biggl(\frac {1}{\sqrt{\sum_{t=1}^{v}(^{v}D_{kt})}} \biggr) \biggr)x_{k}. \end{aligned}$$

Using the Cauchy–Schwarz inequality,

3.5 $$\begin{aligned} \lambda_{1}(G)x_{i}&=\biggl(\frac{1}{\sqrt{nD_{in}}} \biggr) \biggl(\frac{n}{\sqrt {n}} \biggr)x_{k} \end{aligned}$$

3.6 $$\begin{aligned} &= \biggl(\frac{1}{\sqrt{D_{in}}} \biggr)x_{k}. \end{aligned}$$

From Lemmas 2.1 and 2.2, we have

3.7 $$\begin{aligned} \lambda_{1}(G)&\le \sqrt{\frac{1}{\sqrt{D_{in}}}\frac{1}{\sqrt {D_{jn}}}} \end{aligned}$$

3.8 $$\begin{aligned} &\le\frac{1}{^{4}\sqrt{D_{in}D_{jn}}}. \end{aligned}$$

 □

#### Theorem 3.2

*Let* *G* *be a simple*, *connected graph with* *m* *edges and* *n* *vertices*. *Let* $\lambda_{1}, \lambda_{2},\ldots,\lambda_{n}$ *be eigenvalues of the distance*-*sum*-*connectivity matrix* ^*δ*^*X* *and* $E(G)$ *be an energy of* ^*δ*^*X*, *then*

$$\begin{aligned} \lambda_{2}(G) \le \sqrt{\frac{2m(\sqrt{2mn}-d_{1})}{n^{3}d_{1}}- \frac {4m^{2}}{n^{6}d_{1}^{2}}+m}, \end{aligned}$$

*where* $\lambda_{2}$ *is the second greatest eigenvalue of* ^*δ*^*X*.

#### Proof

$\lambda_{2}$ is the second largest eigenvalue of ^*δ*^*X*. Firstly, we show that $\lambda_{1}\geq\frac{2m}{n^{3}d_{1}}$. We know that $(D(G)^{-1}$($^{\delta}\mathit{X}(G)$)$D(G))X=\lambda _{1}(G)X$. So, $\lambda_{1}(G)x_{i}=\sum_{k} ((\delta(i)\delta(j))^{\frac{-1}{2}}\frac {d_{k}}{d_{1}})x_{k}$. Similar to Theorem 3.1, if we take the *i*th equation of this equation, we obtain

3.9 $$\begin{aligned} \lambda_{1}(G)x_{i} &= \Biggl(\sum _{k} \Biggl(\sum_{m=1}^{v} \bigl(^{v}D_{im} \bigr)\sum_{s=1}^{v} \bigl(^{v}D_{js} \bigr) \Biggr)^{\frac{-1}{2}} \frac{d_{k}}{d_{1}} \Biggr)x_{k} \end{aligned}$$

3.10 $$\begin{aligned} &= \biggl(\sum_{k} \biggl(\frac{1}{\sqrt{\sum_{m=1}^{v}(^{v}D_{im})}} \biggr) \biggl(\frac {1}{\sqrt{\sum_{s=1}^{v}(^{v}D_{js})}} \biggr)\frac{d_{k}}{d_{1}} \biggr)x_{k} \end{aligned}$$

Using the Cauchy–Schwarz inequality and calculating the distance matrices of ^*δ*^*X*, we obtain

3.11 $$\begin{aligned} \lambda_{1}(G)x_{i}\geq \biggl(\frac{1}{n\sqrt{n}} \frac{1}{n\sqrt{n}} \biggr) \biggl(\sum_{k} \biggl( \frac{d_{k}}{d_{1}} \biggr) \biggr)x_{k}. \end{aligned}$$

We know that $\sum_{k=1}^{n}=2m$. Hence,

3.12 $$\begin{aligned} \lambda_{1}(G)\geq\frac{2m}{d_{1}n^{3}}. \end{aligned}$$

Secondly, we will show that $\lambda_{2}(G) \le \sqrt{\frac{2m(\sqrt {2mn}-d_{1})}{n^{3}d_{1}}-\frac{4m^{2}}{n^{6}d_{1}^{2}}+m}$.

We know that $\sum_{i=1}^{n}\lambda_{i}=0$ and $\sum_{i=1}^{n}(\lambda _{i})^{2}=2m$. So, $\lambda_{1}+\lambda_{2}=-\sum_{i=3}^{n}\lambda _{i}$. Hence,

$$ \lambda_{2}\leq \vert \lambda_{1} \vert + \Biggl\vert \sum_{i=3}^{n}\lambda_{i} \Biggr\vert . $$

If we take the square of both sides, we obtain

$$ (\lambda_{2})^{2}\leq(\lambda_{1})^{2}+ 2 \vert \lambda_{1} \vert \Biggl\vert \sum _{i=3}^{n}\lambda_{i} \Biggr\vert + \Biggl\vert \sum_{i=3}^{n} \lambda_{i} \Biggr\vert ^{2}. $$

By the Cauchy–Schwarz inequality with the above result, we have

$$\begin{aligned} (\lambda_{2})^{2} &\leq (\lambda_{1})^{2}+ 2 \vert \lambda_{1} \vert \sum_{i=3}^{n} \vert \lambda_{i} \vert +\sum_{i=3}^{n}( \lambda_{i})^{2} \\ &\leq(\lambda_{1})^{2}+ 2 \vert \lambda_{1} \vert \bigl(E(G)- \vert \lambda_{1} \vert - \vert \lambda _{2} \vert \bigr)+2m-(\lambda_{1})^{2}-( \lambda_{2})^{2}. \end{aligned}$$

If we make necessary calculations, we have

$$\begin{aligned} (\lambda_{2})^{2} \leq \vert \lambda_{1} \vert \bigl(E(G) \bigr)- \vert \lambda_{1} \vert ^{2}- \vert \lambda _{1} \vert \vert \lambda_{2} \vert +m. \end{aligned}$$

Since $\lambda_{1}\geq\lambda_{2}$ and $\lambda_{1}\le d_{1}$, then $d_{1}\geq\lambda_{1}\geq\lambda_{2}$. So,

$$\begin{aligned} (\lambda_{2})^{2}&\leq \vert \lambda_{1} \vert \bigl(E(G) \bigr)- \vert \lambda_{1} \vert ^{2}- \vert \lambda _{1} \vert d_{1}+m \\ &\leq \vert \lambda_{1} \vert \bigl(E(G)-d_{1} \bigr)- \lambda_{1}^{2}+m. \end{aligned}$$

Since $E(G)\le\sqrt{2mn}$ and $\lambda_{1}\geq\frac{2m}{n^{3}d_{1}}$, then

$$\begin{aligned} \lambda_{2} &\leq \sqrt{\frac{2m}{n^{3}d_{1}} \bigl(E(G)-d_{1} \bigr)+m- \biggl(\frac {2m}{n^{3}d_{1}} \biggr)^{2}} \\ &\leq\sqrt{\frac{2m(\sqrt{2mn}-d_{1})}{n^{3}d_{1}}-\frac {4m^{2}}{n^{6}d_{1}^{2}}+m}. \end{aligned}$$

 □

### Upper and lower bounds on incidence energy

In the sequel of this paper, we expand bounds under the energy of $^{\delta}\mathit{X}(G)$ with *n*, *D* and $\operatorname{det}(^{\delta}\mathit{X}(G))$.

#### Theorem 3.3

*Let* *G* *be a regular graph of order* *n* *with* *m* *edges*. *Let* $\mathit{IE}(G)$ *be an incidence energy of* $^{\delta}\mathit{X}(G)$ *and* $\sigma_{1}, \sigma _{2},\ldots,\sigma_{n}$ *be singular values of* $^{\delta}\mathit{X}(G)$. *Then*

$$\begin{aligned} \mathit{IE} \bigl(^{\delta}\mathit{X}_{1} \bigr) (G)+\mathit{IE} \bigl(^{\delta}\mathit{X}_{2} \bigr) (G) \le 2\sqrt{\Delta}+ \sqrt{(n-1) \biggl(2\sqrt{2mn}-\frac{4m}{n} \biggr)+2 \biggl\vert \sqrt{2mn}-\frac {2m}{n} \biggr\vert )}. \end{aligned}$$

#### Proof

Let $\sigma_{i}$ and $\sigma_{j}$ be singular values of $(^{\delta }\mathit{X}_{1})(G)$ and $(^{\delta}\mathit{X}_{2})(G)$, respectively. We will use that $\sum_{i=2}^{n}(\sigma_{i})^{2}=\sum_{i=2}^{n}|\lambda _{i}|=E(G)-|\lambda_{1}|$.

By Lemma 2.5,

$$\begin{aligned} \sum_{i=1}^{k}\sigma_{i} \bigl(^{\delta}\mathit{X}_{1}+^{\delta}\mathit {X}_{2} \bigr)\le\sum_{i=1}^{k} \sigma_{i} \bigl(^{\delta}\mathit{X}_{1} \bigr)+\sum _{i=1}^{k}\sigma_{i} \bigl(^{\delta}\mathit{X}_{1} \bigr). \end{aligned}$$

So,

$$\begin{aligned} \sum_{i=2,j=2}^{n}(\sigma_{i}+ \sigma_{j})&\le \sum_{i=2}^{n} \sigma _{i}^{2}+\sum_{i=2}^{n} \sigma_{j}^{2}+2\sqrt{\sum _{i=2}^{n}\sigma _{i}^{2}\sum _{i=2}^{n}\sigma_{j}^{2}} \\ &=E \bigl(^{\delta}\mathit{X}_{1} \bigr)- \lambda_{1}+E \bigl(^{\delta}\mathit {X}_{2} \bigr)- \lambda_{1}+2\sqrt{ \bigl(E \bigl(^{\delta}\mathit{X}_{1} \bigr)-\lambda _{1} \bigr) \bigl(E \bigl(^{\delta} \mathit{X}_{2} \bigr)- \lambda_{1} \bigr)}. \end{aligned}$$

Since $\lambda_{1}\geq\frac{2m}{n}$ and $E(G)\le\sqrt{2mn}$, we get

$$\begin{aligned} \sum_{i=2,j=2}^{n}(\sigma_{i}+ \sigma_{j})\le2\sqrt{2mn}-\frac {4m}{n}+2 \biggl\vert \sqrt{2mn}-\frac{2m}{n} \biggr\vert . \end{aligned}$$

Since $\lambda_{1}\le\Delta$,

$$\begin{aligned} \mathit{IE} \bigl(^{\delta}\mathit{X}_{1} \bigr) (G)+\mathit{IE} \bigl(^{\delta}\mathit{X}_{2} \bigr) (G) &= \sigma_{1}+ \sigma_{1}+\sum_{i=2,j=2}^{n}( \sigma_{i}+\sigma_{j}) \\ &=2\sqrt{\lambda_{1}}+\sqrt{(n-1)\sum _{i=2,j=2}^{n}(\sigma_{i}+\sigma _{j})^{2}}. \end{aligned}$$

Hence,

$$\begin{aligned} \mathit{IE} \bigl(^{\delta}\mathit{X}_{1} \bigr) (G)+\mathit{IE} \bigl(^{\delta}\mathit{X}_{2} \bigr) (G) \le 2\sqrt{\Delta}+ \sqrt{(n-1) \biggl(2\sqrt{2mn}-\frac{4m}{n} \biggr)+2 \biggl\vert \sqrt{2mn}-\frac {2m}{n} \biggr\vert )}. \end{aligned}$$

 □

#### Theorem 3.4

*Let* *G* *be a graph with* *n* *nodes and* *m* *edges*. *Let the smallest and the largest positive singular values* $\sigma_{1}$ *and* $\sigma_{n}$ *of* ^*δ*^*X*, *respectively*, *and* $\operatorname{det}(^{\delta}\mathit{X})$ *be a determinant of the distance*-*sum*-*connectivity matrix* ^*δ*^*X* *of* *G*. *For* $n>1$,

3.13 $$\begin{aligned} E(G)\le\frac{n^{2}}{2(n-1)} \biggl(\frac{1}{^{4}\sqrt{D_{in}D_{jn}}}-\sqrt { \frac{\operatorname{det}(^{\delta}\mathit{X})}{\prod_{i=2}^{n-1}}} \biggr), \end{aligned}$$

*where* $E(G)$ *is the energy of* ^*δ*^*X*.

#### Proof

Suppose $a_{i}=1$ and $b_{i}=\sigma_{i}$, $1\le i\le n$. Apply Theorem 2.3 to show that

3.14 $$\begin{aligned} \sum_{i=1}^{n}1^{2}\sum _{i=1}^{n}\sigma_{i}^{2}- \Biggl( \sum_{i=1}^{n}\sigma _{i} \Biggr)^{2}\le\frac{n^{2}}{4}(\sigma_{n}- \sigma_{1})^{2}. \end{aligned}$$

Thus, it is readily seen that

3.15 $$\begin{aligned} nE(G)\le\frac{n^{2}}{4}(\sigma_{n}-\sigma_{1})^{2}+ \Biggl(\sum_{i=1}^{n}\sigma_{i} \Biggr)^{2}. \end{aligned}$$

By the Cauchy–Schwarz inequality, we can express that

3.16 $$\begin{aligned} nE(G)&\le \frac{n^{2}}{4}(\sigma_{n}-\sigma_{1})^{2}+ \sum_{i=1}^{n}\sigma_{i}^{2} \end{aligned}$$

3.17 $$\begin{aligned} &\le\frac{n^{2}}{4}(\sigma_{n}-\sigma_{1})^{2}+E(G). \end{aligned}$$

Then it suffices to check that

3.18 $$\begin{aligned} E(G)&\le \frac{n^{2}}{4(n-1)}(\sigma_{n}-\sigma_{1})^{2} \end{aligned}$$

3.19 $$\begin{aligned} &\le\frac{n^{2}}{4(n-1)} \bigl(\sigma_{n}^{2}-2 \sigma_{n}\sigma_{1}+\sigma _{1}^{2} \bigr) \end{aligned}$$

3.20 $$\begin{aligned} &\le\frac{n^{2}}{4(n-1)}(\lambda_{n}-2\sqrt{\lambda_{n}} \sqrt{\lambda _{1}}+\lambda_{1}) \end{aligned}$$

3.21 $$\begin{aligned} &\le\frac{n^{2}}{4(n-1)} \biggl(\lambda_{n}-2\sqrt{ \frac{\operatorname{det}(^{\delta}\mathit {X})}{\prod_{i=2}^{n-1}}}+\lambda_{1} \biggr). \end{aligned}$$

Since $\lambda_{1}\geq\lambda_{2}\geq\cdots\geq\lambda_{n}$ and using Theorem 3.1, we obtain

3.22 $$\begin{aligned} E(G)\le\frac{n^{2}}{2(n-1)} \biggl(\frac{1}{^{4}\sqrt{D_{in}D_{jn}}}-\sqrt { \frac{\operatorname{det}(^{\delta}\mathit{X})}{\prod_{i=2}^{n-1}}} \biggr). \end{aligned}$$

 □

### Upper and lower bounds for matching energy

We determine an upper bound for the matching energy applying the Polya–Szego inequality, and we give some results using Maclaurin’s symmetric mean inequality.

#### Theorem 3.5

*Let* *G* *be a connected graph and* $\mathit{ME}(G)$ *be a matching energy of* *G*, *then*

3.23 $$\begin{aligned} \mathit{ME}(G)\geq\frac{\sqrt{8mns_{1}s_{n}}}{ \vert s_{1} \vert + \vert s_{n} \vert }, \end{aligned}$$

*where* $\mu_{i}$ *is the zero of its matching polynomial*.

#### Proof

Let $\mu_{1},\mu_{2},\ldots,\mu_{n}$be the zeros of their matching polynomial. We suppose that $s_{i}=|\mu_{i}|$, where $s_{1}\leq s_{2}\leq\cdots\leq s_{n}$ and $t_{i}=1$, $1\leq i\leq n$. By Theorem 2.4, we obtain

3.24 $$\begin{aligned} \sum_{i=1}^{n} \vert \mu_{i} \vert ^{2}\sum_{i=1}^{n}1^{2} \le\frac{1}{4} \biggl(\sqrt {\frac{ \vert \mu_{n} \vert }{ \vert \mu_{1} \vert }}+\sqrt{\frac{ \vert \mu_{1} \vert }{ \vert \mu _{n} \vert }} \biggr)^{2} \Biggl(\sum_{i=1}^{n} \vert \mu_{i} \vert \Biggr)^{2}. \end{aligned}$$

Since $\sum_{i=1}^{n}\mu_{i}^{2}=2m$,

3.25 $$\begin{aligned} n\sum_{i=1}^{n} \vert \mu_{i} \vert ^{2}\le\frac{1}{4} \biggl(\sqrt{\frac{ \vert \mu_{n} \vert + \vert \mu _{1} \vert }{ \vert \mu_{1}\mu_{n} \vert }} \biggr)^{2} \Biggl(\sum_{i=1}^{n} \vert \mu_{i} \vert \Biggr)^{2}. \end{aligned}$$

It is easy to see that

3.26 $$\begin{aligned} \mathit{ME}(G)\geq\frac{\sqrt{8mn \vert \mu_{1}\mu_{n} \vert }}{ \vert \mu_{1} \vert + \vert \mu_{n} \vert }. \end{aligned}$$

We can assume that the maximum $|\mu_{i}|$ is $s_{n}$ and the minimum $|\mu_{i}|$ is $s_{1}$. So the bound can be sharpened, that is,

3.27 $$\begin{aligned} \mathit{ME}(G)\geq\frac{\sqrt{8mns_{1}s_{n}}}{ \vert s_{1} \vert + \vert s_{n} \vert }. \end{aligned}$$

 □

#### Corollary 3.6

*Let* *G* *be a* *k*-*regular graph*. *Then*

3.28 $$\begin{aligned} \mathit{ME}(G)\geq\frac{2nk\sqrt{s_{1}}}{ \vert s_{1} \vert +k}. \end{aligned}$$

#### Proof

Since *G* is a *k*-regular graph, we can take $s_{n}=k$ and $2m=nk$. By Theorem 3.5,

3.29 $$\begin{aligned} \mathit{ME}(G)\geq\frac{\sqrt{4n^{2}k^{2}s_{1}}}{ \vert s_{1} \vert +k}. \end{aligned}$$

Hence,

3.30 $$\begin{aligned} \mathit{ME}(G)\geq\frac{2nk\sqrt{s_{1}}}{ \vert s_{1} \vert +k}. \end{aligned}$$

 □

#### Theorem 3.7

*Let* *G* *be a connected graph with n vertices and m edges*. *Then*

3.31 $$\begin{aligned} \mathit{ME}(G)\geq n\sqrt{\frac{2m}{n-1}} \end{aligned}$$

*if and only if* $\mu_{1}=\mu_{2}=\cdots=\mu_{n}$.

#### Proof

Let us consider $s=n$ and $x_{i}=|\mu_{i}|$ for $i=1,2,\ldots,n$. Setting that in Lemma 2.6, we get

3.32 $$\begin{aligned} M_{1}=\frac{\sum_{i=1}^{n} \vert \mu_{i} \vert }{n}=\frac{\mathit{ME}(G)}{n}. \end{aligned}$$

Also,

3.33 $$\begin{aligned} M_{2}=\frac{1}{n(n-1)}\sum_{i=1}^{n} \sum_{j=1,j\neq i}^{n} \vert \mu_{i} \vert \vert \mu_{j} \vert . \end{aligned}$$

Since $\sum_{i=1}^{n}\mu_{i}^{2}=2m$ and $\sum_{i< j}|\mu_{i}||\mu _{j}|=-m$, then

3.34 $$\begin{aligned} M_{2}=\frac{1}{n(n-1)}\sum_{i=1}^{n}m^{2}= \frac{m^{2}}{n(n-1)}. \end{aligned}$$

We know that $M_{1}\geq M_{2}^{\frac{1}{2}}$. So,

3.35 $$\begin{aligned} \mathit{ME}(G)\geq\frac{mn}{n-1}. \end{aligned}$$

The above equality holds if and only if $\mu_{1}=\mu_{2}=\cdots=\mu_{n}$. □

#### Theorem 3.8

*Let* *G* *be a connected graph with n vertices and m edges*. *Then*

3.36 $$\begin{aligned} \mathit{ME}(G)\leq^{2\ell}\sqrt{(2m)^{\ell}-n(n-1)\prod _{i=1}^{n} \vert \mu _{i} \vert ^{\frac{2\ell}{n}}} \end{aligned}$$

*if and only if* $\mu_{1}=\mu_{2}=\cdots=\mu_{n}$.

#### Proof

Let us consider $s=n$ and $x_{i}=|\mu_{i}|^{\ell}$ for $i=1,2,\ldots,n$. By Lemma 2.6, we determine

3.37 $$\begin{aligned} M_{2}&=\frac{1}{n(n-1)}\sum_{i=1}^{n} \sum_{j=1,j\neq i}^{n} \vert \mu _{i} \vert ^{\ell} \vert \mu_{j} \vert ^{\ell} \end{aligned}$$

3.38 $$\begin{aligned} &=\frac{1}{n(n-1)} \Biggl( \Biggl(\sum_{i=1}^{n}| \mu_{i}|^{\ell}| \Biggr)^{2}-\sum _{i=1}^{n} \bigl( \vert \mu_{j} \vert ^{\ell} \bigr)^{2} \Biggr). \end{aligned}$$

Using the Cauchy–Schwarz inequality, we have

3.39 $$\begin{aligned} M_{2}\leq\frac{1}{n(n-1)} \Biggl( \Biggl(\sum _{i=1}^{n} \vert \mu_{i} \vert ^{\ell} \Biggr)^{2}- \Biggl(\sum_{i=1}^{n} \vert \mu_{j} \vert \Biggr)^{2\ell} \Biggr). \end{aligned}$$

It is clear that the above equality gives

3.40 $$\begin{aligned} M_{2}=\frac{1}{n(n-1)} \bigl((2m)^{\ell}- \bigl(\mathit{ME}(G) \bigr)^{2\ell} \bigr). \end{aligned}$$

Thus, it is pointed out that $(\mathit{ME}(G))^{2\ell}\leq(2m)^{\ell}-n(n-1)M_{2}$. Since $M_{2}^{\frac {1}{2}}\geq M_{n}^{\frac{1}{n}}$ and $M_{n}=\prod_{i=1}^{n}|\mu _{i}|^{\ell}$, then

3.41 $$\begin{aligned} \bigl(\mathit{ME}(G) \bigr)^{2\ell}\leq(2m)^{\ell}-n(n-1) \Biggl(\prod _{i=1}^{n} \vert \mu_{i} \vert ^{\ell } \Biggr)^{\frac{2}{n}}. \end{aligned}$$

Hence,

3.42 $$\begin{aligned} \mathit{ME}(G)\leq^{2\ell}\sqrt{(2m)^{\ell}-n(n-1)\prod _{i=1}^{n} \vert \mu _{i} \vert ^{\frac{2\ell}{n}}}. \end{aligned}$$

The above result holds if and only if $\mu_{1}=\mu_{2}=\cdots=\mu_{n}$. □

## Conclusions

The main goal of this work is to examine distance-sum-connectivity matrix ^*δ*^*X*. We find some upper bounds for the distance-sum-connectivity matrix of a graph involving its degrees, its edges, and its eigenvalues. We also give some results for the distance-sum-connectivity matrix of a graph in terms of its energy, its incidence energy, and its matching energy.
